# Oral Anticoagulant Therapy: When What Should Not Be Is and What Should Be Is Not

**DOI:** 10.19102/icrm.2019.100803

**Published:** 2019-08-15

**Authors:** James A. Reiffel

**Affiliations:** ^1^Department of Medicine, Division of Cardiology, Electrophysiology Section, Columbia University, New York, NY, USA

**Keywords:** Anticoagulation, atrial fibrillation, percutaneous coronary intervention

In the current issue of *The Journal of Innovations in Cardiac Rhythm Management*, Rusia et al. report on anticoagulation patterns in patients with atrial fibrillation (AF) who underwent percutaneous coronary intervention (PCI) at two academic institutions in California between 2008 and 2016.^1^ Their retrospective analysis was designed to identify factors that may have influenced the appropriate use of oral anticoagulants (OACs) in this population of patients who also required antiplatelet therapy following their PCI procedure. Unfortunately, of the 4,648 patients who underwent PCI, only 211 also had AF and an indication for OAC therapy, thus making the study’s dataset quite small and therefore perhaps limiting the ability for adequate interpretation. Moreover, of the 211 patients, only 105 who met the indications for OAC treatment actually received OAC therapy after their PCI and, of these, only six received a direct-acting OAC (DOAC), with the rest receiving warfarin. Accordingly, given the limited use of DOACs, the years for which observations were made, and the differences between guidelines from this period and our more current ones regarding the combined use of OACs and antiplatelet agents in AF patients following PCI,^2–5^ it is possible that the current relevance of the observations could be questioned. Additionally, the report only refers to “community physicians” and provides no data as to whether the prescribing habits differed by generalists or specialists.

Nonetheless, however, several interesting observations were made in this study that may have relevance to the broader population of similar patient groups and/or similar community physician groups. First, there was no significant relationship between discharge on OAC therapy and HAS-BLED score. This would be a highly encouraging finding if it reflected knowledge rather than fear. Thromboembolic strokes associated with AF are more apt to be fatal or debilitating in comparison with bleeding events on OAC therapy (excluding the very small number associated with intracranial bleeding on a DOAC). Thus, fear of bleeding in most patients should not prevent the consideration of and usually use of OAC therapy in AF patients identified as being at an increased risk for thromboembolism.^6^ Unless the HAS-BLED score is high and the CHA_2_DS_2_-VASc score is low, the latter rather than the former should be the deciding factor in whether or not to prescribe an OAC. The prevention of stroke should usually take the highest priority. Rusia et al.^1^ were aware of this (although their surveyed hospital physicians may not have been) when they wrote in their report: “a reduced stroke risk generally far outweighs the risk of major hemorrhage while on anticoagulation and most concerns about bleeding are generally unfounded. Simply put, the oldest patients with AF are those who have the highest risk of stroke and the most to gain from anticoagulation.” Second, and disturbing rather than encouraging, Rusia et al.^1^ found no statistical association between discharge on an OAC and CHA_2_DS_2_-VASc score, with a worrisome trend occurring in the opposite direction. A higher CHA_2_DS_2_-VASc score indicates a greater risk for thromboembolism in AF and should be associated with a higher use of OACs. Strikingly, patients who were younger than 65 years of age were much more likely to receive triple therapy (56% versus 33%) or any OAC (69% versus 41%) than older patients. Notably, while those patients aged 65 years and older had higher HAS-BLED scores (2.8 versus 2.4), they also had higher CHA_2_DS_2_-VASc scores (4.4 versus 3.2). Taken together, these observations may suggest that this group of physicians feared bleeding in all subjects somewhat similarly (regardless of HAS-BLED score) and did so to a degree that they avoided OAC or triple therapy in the subjects who needed such medications the most. While it is true that older patients with more comorbidities are more likely to bleed, they are also the most likely to suffer thromboembolism and gain the most from OAC therapy. Clearly, the paper suggests an ongoing need for education in this arena.

Not uncommonly, physicians have used a fear of more elderly patients falling and suffering a head or other major injury as a reason to avoid anticoagulation. While we have no way of knowing from the Rusia et al.^1^ report whether this was the case in their surveyed institutions, there is literature-reported data to indicate that this concern is excessive. In 2012, Donze et al. examined 515 consecutive adult medical patients discharged on an OAC.^7^ Of these, 308 were deemed by their physicians to be at a high risk of falls, while 207 were deemed to not be at a high risk for such. In these two groups, there were ultimately 8.0 falls per 100 patient-years and 6.8 falls per 100 patient-years, respectively (p = 0.64). In the total population, there were 35 major bleeds (7.5 per 100 patient-years), with only three associated with a fall (0.6 per 100 patient-years). Thus, major bleeds were not statistically associated with falls, and physician determination of fall risk was not very accurate. In a more recent study^8^ that examined outcomes of patients on apixaban (not discussed in the Rusia et al. report^1^) in comparison with warfarin, stratified by a history of falls or absence thereof, although patients with a history of falls had higher rates of major bleeding (hazard ratio: 1.39; p = 0.020), including intracranial bleeding (hazard ratio: 1.87; p = 0.044), in those with a history of falls, there were five subdural bleeds among 367 patients on warfarin and zero among 386 patients on apixaban. Importantly, patients with a history of falls were more likely to be female and to have a history of dementia, cerebrovascular disease, depression, diabetes, heart failure, osteoporosis, fractures, higher CHA_2_DS_2_-VASc scores, and higher HAS-BLED scores. The message here is not to focus on falls but rather that patients who fall are more likely to have the very factors that indicate an increased risk of thromboembolism in AF and therefore benefit from OAC therapy—especially considering the newer DOACs that were not available during the time frame of the Rusia et al. report.^1^

Finally, in dealing with patients who have undergone PCI and who require antiplatelet therapy postprocedure but who also have AF and require an OAC, current recommendations suggest not to avoid OAC and also not to avoid dual or triple therapy. Rather, they deal with the specifics of the antiplatelet agents to be used, the duration of triple versus dual therapy, and methods to reduce the risk of bleeding. OACs should be used if the AF risk profile indicates a need; DOACs are preferable to warfarin, including or especially when combined with antiplatelet agents; and antiplatelet agents should be used as per post-PCI indications. All physicians who are involved with the management of such patients should become familiar with these current recommendations and guidelines and avoid the selection errors reported by Rusia et al.^1^ These references include the 2019 American Heart Association/American College of Cardiology/Heart Rhythm Society (AHA/ACC/HRS) focused update of the 2014 AHA/ACC/HRS guidelines for the management of patients with AF^2^; the recent article titled “Antithrombotic Therapy in Patients with Atrial Fibrillation Treated with Oral Anticoagulation Undergoing Percutaneous Intervention: A North American Perspective”^3^; the 2018 Joint European Consensus Document on the Management of Antithrombotic Therapy in Atrial Fibrillation Patients Presenting with Acute Coronary Syndrome and/or Undergoing Percutaneous Cardiovascular Interventions^4^; and the 2018 Focused Update of the Canadian Cardiovascular Society Guidelines for the Management of Atrial Fibrillation.^5^

Hence—when what should not be is and what should be is not. What should not be is the avoidance of OAC usage in AF patients with a high thromboembolic risk or the avoidance of appropriate dual or triple therapy per guidelines-indicated regimens in AF patients who undergo PCI. What should be is the appropriate use of such therapies coupled with their appropriate duration and the management of any underlying comorbidities, social situations, adverse habits, and the like that may minimize the risk of bleeding while using these life-saving, thrombosis-reducing therapies.
